# Clinical safety and efficacy of a hydrophilic acrylic intraocular lens in a real-world population: a 1-year follow-up retro-prospective study

**DOI:** 10.1186/s12886-020-01493-y

**Published:** 2020-06-11

**Authors:** Björn Johansson, Ana C. S. Daniel, Claudia Herbers, Matthias Gerl, Florian T. A. Kretz

**Affiliations:** 1 Division of Sensory Organs and Communication, Department of Biomedical and Clinical Sciences, Linköping University, SE58183, Linköping, Sweden; 2 Augentagesklinik Rheine, Osnabrücker Straße 233-235, 48429 Rheine, Germany; 3 Augenklinik Ahaus, Am Schlossgraben 13, 48683 Ahaus, Germany

**Keywords:** Monofocal, Intraocular lens, IOL, Real-world, Visual outcome, Ocular pathology, Human

## Abstract

**Background:**

This multicentre, retro-prospective real-world study evaluated the visual, refractive and safety outcomes of a monofocal lens 1 year after implantation in cataract patients with or without pre-existing ocular pathologies.

**Methods:**

Records from 4 centres in Germany and Sweden were reviewed to select eyes with aged-related cataracts, having undergone crystalline lens extraction by phacoemulsification and implantation of a CT ASPHINA 409 IOL. Preoperative, 1-month and 3-month postoperative data was collected retrospectively. In addition, included patients attended a prospective visit 12 months or later after surgery. The examination included: monocular uncorrected (UDVA) and corrected distance visual acuity (CDVA), subjective refraction, slit-lamp examination, optical biometry, intraocular pressure (IOP), endothelial cell count and postoperative complications.

**Results:**

282 eyes, including 94 with pre-existing ocular pathologies, were analysed. Twelve months after the surgery, 95% of eyes achieved monocular CDVA equal or better than 0.3 logMAR, mean postoperative CDVA was 0.06 ± 0.17 logMAR, and mean UDVA 0.31 ± 0.29 logMAR. Visual acuity outcomes were better in eyes with no pre-existing ocular pathologies, but both groups showed a statistically significant improvement after surgery compared with preoperative values (*p* ≤ 0.002). The mean sphere and spherical equivalent values also improved significantly postoperatively (*p* = 0.003). Overall, 62.1% of eyes had spherical equivalent within ±0.5 D and 80.9% within ±1.0 D. The IOL was stable in the capsular bag as demonstrated by tilt and decentration measurements. IOP, corneal status, and endothelial cell count values were in the normal range. Nd:YAG treatment was performed on 9.9% of the eyes.

**Conclusion:**

The implantation of the monofocal CT ASPHINA 409 IOL was beneficial to restore vision in eyes with or without concomitant ocular pathology such as macular degeneration, glaucoma, Sicca syndrome, epiretinal membrane, cornea guttata, or amblyopia. Good to excellent long-term visual and refractive outcomes, and a low rate of complications in both healthy and pathological eyes were found 12 months after the surgery.

**Trial registration:**

Trial registered on under the identification NCT03145103 (date of registration 9 May 2017).

## Background

Cataract afflicts millions of people worldwide and is still today the major cause of blindness. It is estimated that roughly 20 million people are blind because of bilateral cataract worldwide [1]. In addition to the reduction in visual acuity, cataract has been correlated with depression, decreased quality of life for the patients and their caretakers and increased mortality rates in the elderly [1].

Many models of intraocular lenses (IOLs) are currently available on the market to adapt to the widest possible population and fulfil, when possible, the exact postoperative desires of the patients. Premium IOLs, including toric, multifocal and more recently extended depth of focus lenses and accommodative IOLs [2] restore distance visual function, and can also mimic more closely the optimal performance of the eye.

Despite this technological progress, monofocal IOLs are still the most widely implanted lenses worldwide. Reasons for their popularity include lower price, patient preference [3], patient natural sensitivity to glare and halos [3], or the presence of coexisting ocular conditions [4].

The CT ASPHINA 409 IOL is an aspheric, aberration neutral, monofocal lens designed for implantation through an incision of 1.8 mm or more. The design of the lens features four-point plate haptics and one-piece construction from a hydrophilic acrylic material with hydrophobic surface properties. This lens is commercialised in Europe since 2006. This retro-prospective study was designed to evaluate the visual and refractive outcomes as well as the safety of the CT ASPHINA 409, 1 year after implantation in real-world cataract patients.

## Methods

### Patient population

This multicentre, retro-prospective, clinical study was conducted at 4 sites in Germany and Sweden between August 2017 and June 2018. The protocol was reviewed and approved by the Ethics Committee of each site, followed the tenets of the Declaration of Helsinki and fully complied with the International Conference on Harmonization and Good Clinical Practice guidelines. All patients provided a written informed consent prior to enrolment. This trial was registered before the study began with ClinicalTrials.gov, number NCT03145103.

The study included patients who had previously undergone uncomplicated age-related cataract extraction and in-the-bag implantation of a CT ASPHINA 409 IOL in one eye (retrospective patient selection). Inclusion and exclusion criteria were strictly defined to only take into consideration preoperative aspects. Patients for whom CDVA was not available preoperatively or patients with preoperative CDVA better than 0.3 logMAR were excluded from the study. All the remaining patients were invited to attend a postoperative visit at least 12 months after the implantation (prospective data collection).

The primary objective of the trial was to compare the postoperative rate of eyes with CDVA 0.3 logMAR or better to the threshold value given in ISO 11979-7:2014 (92.5%). The minimal recommended number of subjects to achieve this objective was 282.

### Surgery

Surgeries were performed by experienced surgeons with at least 3 years of practice according to their normal protocol. Standard single plane, self-sealing clear corneal incision, capsulorhexis and conventional phacoemulsification was used in all cases. Phacoemulsification was performed through a 1.8 mm incision in 3.9% of cases, a 2.0 mm incision in 48.9% of cases, a 2.2 mm in 2.5% of cases or a 2.7 mm in 44.7% of cases. After a continuous circular capsulorhexis of about 5.5 mm and hydrodissection, the cataract was removed by phacoemulsification with stop-and-chop technique. The CT ASPHINA 409 IOL was subsequently implanted in the capsular bag using a qualified injector in combination with a viscoelastic device. At the end of the surgery, any residual ophthalmic viscoelastic device was thoroughly removed by irrigation, and side ports and main incision were sealed by hydration. Postoperative treatment and medication were given according to the routine procedure in each centre.

The IOL specifications are summarized in Table 1.

**Table 1:**
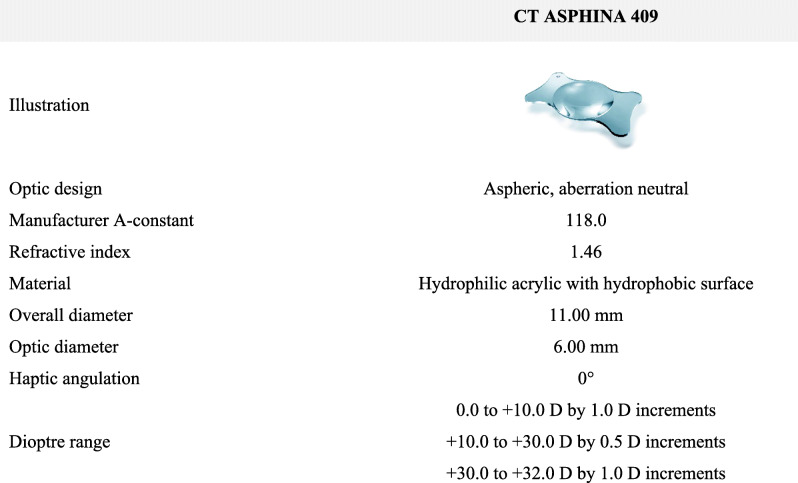
Characteristics of the CT ASPHINA 409 IOL

### Retrospective data collection

Preoperative, surgery-related, 1- and 3-month postoperative data was collected retrospectively when available. Preoperative data collection included medical history, relevant concomitant pathologies and treatments, monocular uncorrected and corrected distance visual acuities (UDVA and CDVA), subjective refraction, biometry, intraocular pressure, endothelial cell count and slit-lamp examination results. Surgery-related data included intraoperative complications, IOL power and expected postoperative refraction. Finally, postoperative monocular visual acuity, and refraction outcomes were collected 1 and 3 months after the surgery when available along with any complications.

### Prospective, postoperative examinations

During the postoperative visit planned at 12 months or more, the patients received a detailed ophthalmologic examination including UDVA and CDVA, subjective refraction (sphere, cylinder and spherical equivalent), slit-lamp examination of the anterior and posterior segment (corneal status, inflammatory reaction, fundus examination, lens opacity, IOL centration, tilt and dislocation), optical biometry (IOLMaster, Carl Zeiss Meditec AG, Jena, Germany), intraocular pressure and endothelial cell count. Adverse events, including posterior capsule opacification (PCO) and neodymium-doped yttrium aluminium garnet (Nd:YAG) rates, were recorded during the follow-up period.

### Statistical analysis

Statistical analysis was performed using SAS Version 9.4 (SAS Institute Inc., Cary, USA). Quantitative endpoints are presented as the mean ± standard deviation and range (minimum; maximum). Qualitative endpoints are presented in terms of number and percentage of each modality and number of patients. In all cases, a *p*-value less than 0.05 was considered as statistically significant.

For quantitative endpoints, Student’s t-test for parametric test or Wilcoxon test for non-parametric tests were used. For qualitative endpoints, standard Chi-square test for parametric tests or Fisher exact test or Cochran-Mantel-Haenszel test for non-parametric tests were used.

## Results

### Patient characteristics

A total of 282 patients (safety population) were included into the study after signing the informed consent; they attended the postoperative visit after a mean follow-up of 510 ± 114 days. Incision size ranged from 1.8 mm to 2.7 mm (mean 2.31 ± 0.35 mm). The preoperative characteristics are given in Table 2.

**Table 2:** Preoperative patient characteristics

	n^a^	Mean ± SD	Range
**Patient characteristics**			
Age (years)	282	73 ± 7.7	49; 91
Gender (%, men/women)	282	46.5 / 53.5	
Axial length (mm)	280	23.82 ± 1.43	21.47; 33.83
Anterior chamber depth (mm)	277	3.16 ± 0.42	1.91; 4.82
IOL power (D)	282	19.89 ± 3.24	1.0; 27.5
Formula	279	Haigis: 52.7% SRK/T: 47.0% Holladay: 0.4%	
Expected postoperative spherical equivalent (D)	278	−0.43 ± 0.97	−6.27; 2.84
**Refraction**			
Sphere (D)	149	−0.65 ± 3.14	−16.0; 6.5
Cylinder (D)	148	−0.87 ± 0.72	−3.5; 0
Spherical equivalent (D)	148	−1.07 ± 3.15	−16.0; 6.0
**Visual acuity**			
UDVA	53	0.84 ± 0.45	0.2; 2.3
CDVA	280	0.48 ± 0.28	0.1; 2.3

^a^ Due to the retrospective nature of the trial, not all individual preoperative values were available. In particular, subjective refraction and UDVA were not routinely measured preoperatively in all sites

Amongst the selected eyes, 2 subpopulations were defined. The “healthy eye” group that included 188 eyes (66.66% of overall population) with cataract but without any ocular pathology that could potentially affect visual acuity. And the “pathological eye” group that included 94 eyes (33.3% of overall population) with at least one concomitant pathology potentially affecting visual acuity. The main pathologies reported were: macular degeneration (42.6%), glaucoma (22.3%), Sicca syndrome (11.7%), epiretinal membrane (9.6%), cornea guttata (9.6%), hypertony (9.6%) and amblyopia (4.3%). In addition, 10.6% of the eyes had other retinal pathologies (e.g. diabetic retinopathy, retinal scar), 8.5% had other various macular abnormalities (e.g. atrophy or oedema), 7.5% had corneal abnormalities (e.g. scar, verticillata, vacuoles) and 5.1% had other various eye abnormalities (e.g. shallow anterior chamber or excavated papilla).

**Fig. 1:**
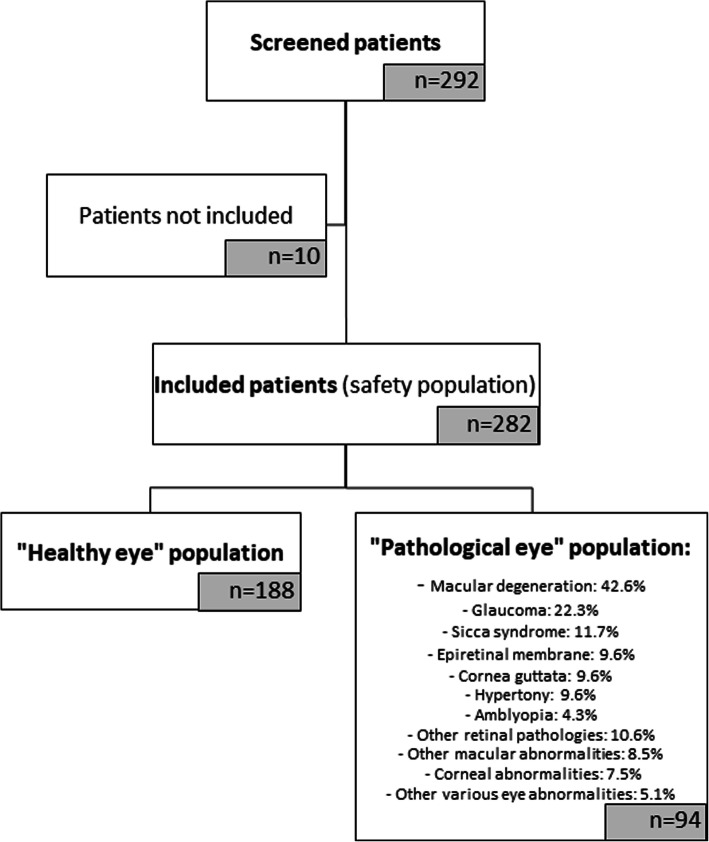
Flow diagram of the clinical trial, showing the 3 populations analysed: the safety population, the “healthy eye” population and the “pathological eye” population

Details of the analysed data sets are given in Fig. 1.

### Visual acuity

**Fig. 2:**
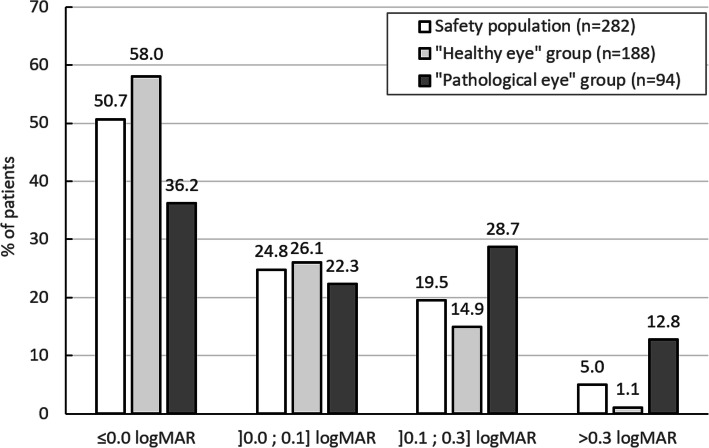
Distribution of monocular corrected distance visual acuity (CDVA) at 12 months

**Fig. 3:**
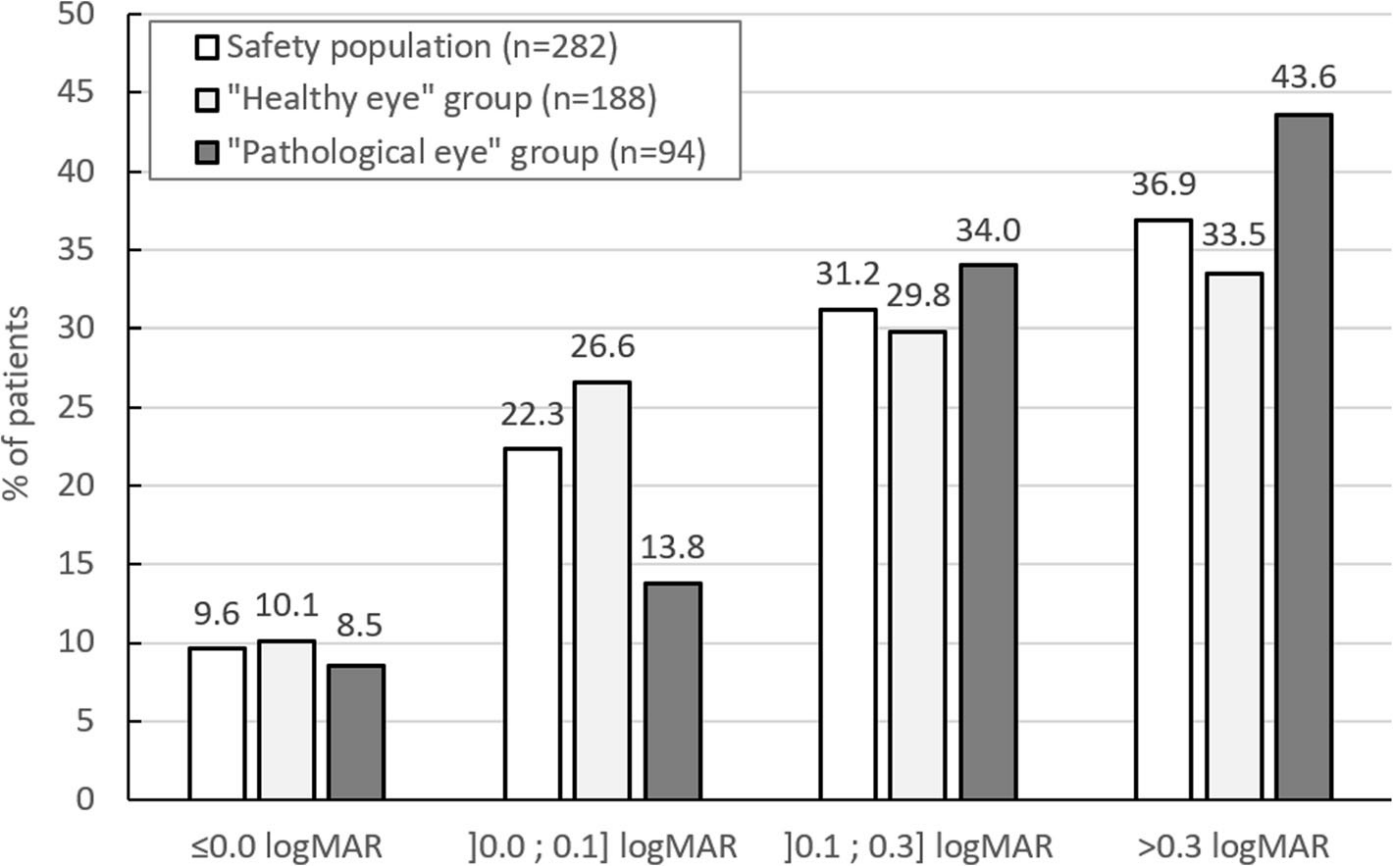
Distribution of monocular uncorrected distance visual acuity (UDVA) at 12 months

Monocular CDVA and monocular UDVA per visual acuity class are shown in Fig. 2 and Fig. 3 for the three populations (safety population, “healthy eye” population and “pathological eye” population).

At 12 months, 95% (CI 92.5%; 97.5%) of the eyes in the safety population had monocular CDVA equal or better than 0.3 logMAR. The 14 eyes that did not reach this visual acuity level were very often multi-pathological eyes. An exhaustive list of the eye-related pathologies likely to have contributed to the reduced visual acuity outcome is presented in Table 3. Amongst these eyes, 5 had a postoperative gain in visual acuity including 4 who gained 1 line or more. Four eyes experienced no change in visual acuity and 5 experienced a loss in visual acuity. Mean postoperative CDVA in the safety population was 0.06 ± 0.17 logMAR (*p* < 0.001 versus preoperative value). There was no statistically significant change in CDVA during the follow-up (*p* = 0.759).

**Table 3:** List of the eye-related pathologies experienced by the 14 patients from the safety population not reaching monocular CDVA equal or better than 0.3 logMAR

Case number	CDVA (logMAR)		Factors contributing to CDVA > 0.3 logMAR
	Preop.	12 months	
1	0.4	0.6	Diabetic macular oedema; diabetic proliferative retinopathy; clinically significant PCO requiring Nd:YAG
2	1.0	0.92	Amblyopia
3	0.4	0.4	No distinct ocular disorders despite diabetes; clinically significant PCO requiring Nd:YAG
4	2.3	0.7	Cornea guttata; macular atrophy; early stage band keratopathy
5	1.3	1.3	Retinal scar (geographic atrophy); amblyopia
6	0.4	0.5	Dry macular degeneration; geographic atrophy
7	0.6	0.6	Amblyopia; macular traction; retinoschisis
8	0.4	0.5	Dry macular degeneration; adult vitelliform maculopathy
9	0.4	1.0	Cornea guttata; ocular hypertension requesting surgical intervention (trabeculectomy) and leading to hyperaemia and hyphema
10	0.4	0.4	Glaucoma; dry macular degeneration; drusen macula; glaucomatous papilla; amblyopia
11	0.52	0.34	Epiretinal membrane
12	0.3	0.4	No distinct ocular disorders despite diabetes mellitus; clinically significant PCO requiring Nd:YAG
13	0.6	0.32	Age-related macular degeneration; transition from drusen age-related macular degeneration to neovascular wet age-related macular degeneration; corneal erosion; clinically significant PCO
14	0.7	0.38	Slight age-related macular degeneration with drusen; vitreous detachment; clinically significant PCO requiring Nd:YAG

In the “healthy eye” group, 98.9% (CI 100%; 94.7%) of the eyes reached monocular CDVA equal or better than 0.3 logMAR and the mean CDVA at 12 months was 0.02 ± 0.11 logMAR. In the “pathological eye” group, 87.2% (CI 93.9%; 80.4%) of eyes reached monocular CDVA equal or better than 0.3 logMAR and the mean CDVA at 12 months was 0.15 ± 0.24 logMAR. In these 2 subpopulations, CDVA improved significantly postoperatively compared with baseline values (*p* < 0.001).

Mean UDVA values at 12 months were the following: 0.31 ± 0.29 logMAR in the safety population, 0.29 ± 0.30 logMAR in the “healthy eye” group, and 0.36 ± 0.27 logMAR in the “pathological eye” group. The improvement was statistically significant in the three groups compared with preoperative values (*p* ≤ 0.002).

### Refraction

In the overall population the mean sphere and spherical equivalent values improved significantly: from − 0.65 ± 3.14 D at the baseline visit to 0.11 ± 1.10 D at the end of the follow-up (*p* = 0.003) for the sphere and from − 1.07 ± 3.15 D to − 0.29 ± 1.09 D, *p* = 0.003 for the spherical equivalent. Over the same period, there was a slight but non-significant improvement of the cylinder (from − 0.87 ± 0.72 D to − 0.81 ± 0.63 D, *p* = 0.068).

Between the 1-month follow-up and the end of the study, there was no detectable refractive shift (*p* ≥ 0.470 for sphere, cylinder and spherical equivalent).

Twelve months postoperatively, 62.1% of eyes had spherical equivalent within ±0.5 D and 80.9% within ±1.0 D. In terms of predictability, the mean difference between expected refraction and the postoperative spherical equivalent at 12 months was − 0.15 ± 0.51 D (*p* < 0.001). There was no difference in predictability between the 2 most used formulas (Haigis; SRK/T). At the 12-month follow-up, 67.0 and 93.0% of patients achieved a final spherical equivalent within ±0.5 D and ± 1 D respectively of the predicted figure.

### Safety

In the overall population, 277 (98.2%) of the IOLs were centred. The maximum horizontal or vertical decentration in the 5 (1.8%) remaining eyes was ≤1 mm. Tilt was noticed in 1 eye only and was also minimal (1 degree).

Overall, intraocular pressure, corneal status, inflammatory reaction and endothelial cell count were all normal at the 12-month follow-up. More specifically, intraocular pressure decreased from 16.16 ± 3.40 mmHg to 13.52 ± 3.31 mmHg between the preoperative and the 12-month visits (*p* < .001). Corneal opacification was observed in only 1 eye (0.4%) in a patient who was also diagnosed with band keratopathy and cornea guttata. No signs of inflammatory cells, fibrin or flare were observed at 12 months. And the endothelial cell count was 2096 ± 483 cells/mm^2^ at the end of the follow-up.

The main postoperative ocular events observed at the 12-month follow-up were the following: 13 (4.6%) patients reported dry eye sensation, 6 (2.1%) patients developed dry age-related macular degeneration, 6 (2.1%) had vitreous detachment, 5 (1.8%) had cystoid macular oedema, 2 (0.7%) had iritis and 1 (0.4%) had corneal oedema. Amongst these events, only 3 cases of cystoid macular oedema were described as ‘likely’ or ‘certainly’ related to the surgery and no event was described as related to the IOL. No IOL dislocation or explantation occurred during the study. Finally, 27 (9.9%) patients received a Nd:YAG treatment during the follow-up,

## Discussion

This study evaluated the real-world performance and safety of the monofocal, aspheric CT ASPHINA 409 IOL 1 year after implantation in eyes with age-related cataract.

In terms of performance, the CT ASPHINA 409 IOL fulfilled ISO 11979-7:2014 criteria, with 95% of the eyes reaching a monocular CDVA equal or better than 0.3 logMAR. This percentage even increased to 98.9% when considering the “healthy eye” group. This outcome is comparable to the data communicated by Alcon with a similar lens, the monofocal, aspheric SA60WF IOL: 96.9% in a population of 129 eyes [5]. The primary outcomes of this trial also compare favourably with the real-world data published from the EUREQUO database [6]. Data on more than 368,000 cataract extractions were analysed, including 25.6% eyes with an ocular comorbidity, and 90% of cases achieved CDVA of 0.3 logMAR. Logistic regression analysis demonstrated that ocular comorbidity and postoperative complications were the two most decisive variables for good clinical outcomes [6].

Mean CDVA (0.06 ± 0.17 logMAR) and UDVA (0.31 ± 0.29 logMAR) at 12 months were slightly higher than values previously reported in prospective clinical trials with similar IOLs, presumably on healthy eyes [7, 8]. However, the outcomes of this study are similar to real-world CDVA values reported by EUREQUO (0.057 ± 0.26 logMAR) [9], and the Royal College of Ophthalmologists (0.16 ± 0.30 logMAR) [10]. These two studies also included eyes with concomitant ocular pathologies (respectively 27.3 and 36.9% of the studied population).

In this study, good refractive predictability was achieved with 67 and 93.0% of eyes respectively within ±0.5 D and ± 1 D of the predicted value. These values were consistent with benchmark values previously reported [11, 12] and slightly lower than the figures reported in the EUREQUO study (93.8% of eyes within ±1 D and 73.7% within ±0.5 D) [9]. The mean difference between the expected refraction and the postoperative spherical equivalent was very moderate (− 0.15 ± 0.51 D) indicative of a high accuracy of the predicted refraction. The targeted spherical equivalent was − 0.43 ± 0.97 D and the achieved value at 12 months − 0.29 ± 1.09 D. Although this difference was statistically significant (*p* < 0.01), its clinical significance was questionable as the value was very close to zero and well below a quarter of a dioptre.

With regards to safety aspects, the overall number of postoperative ocular events was low. Furthermore, they appear to be related to the surgical procedure in general rather than to the IOL itself and do not outweigh the benefits of the procedure in the studied eye.

There are several limitations in this study that could be addressed in future research. First, the partly retrospective study design makes risk for selection bias a concern. To avoid this, participants were only enrolled according to the objective inclusion and exclusion criteria defined in the protocol and enrolment logs were externally monitored. As seen in this paper, it resulted in a significant number of patients with comorbidities reflecting a true real-life scenario. In general, our preoperative visual acuity was worse than in other clinical trials evaluating monofocal IOLs and the sample size by far greater [13–15]. Another limitation of this study regards safety aspects and the absence of data available for preoperative endothelial cell count. Still as a reduction over time is physiological and the surgical procedure itself plays a much greater role in posterior-chamber IOLs we do not feel that this data would add further value to the study. Finally, we describe in this paper the outcomes of a retro-prospective study that was not initially intended to include patients with concomitant ocular pathologies. The data from this subpopulation of 94 eyes is nonetheless important to report as such patients undergo cataract extraction and IOL implantation with increasing frequency.

Ocular co-pathology was found in 33.3% of the patients, which is consistent with rates previously reported in real-world analyses [9, 10]. The nature of the co-pathologies was also consistent with these previous reports, with age-related macular degeneration, glaucoma, diabetic retinopathy and amblyopia being the most frequently recorded.

Postoperative corrected and uncorrected visual outcomes were slightly inferior in the group of eyes with concomitant pathology compared with the population of healthy eyes. However, the benefit/risk ratio for both populations was still favourable. Deciding to implant an IOL in an eye with concomitant pathology is often challenging; the stability of the disease, its expected progression over time, and the anticipated usefulness of the treatment are aspects that should be taken into consideration. The results of this study are consistent with previous publications that showed that phacoemulsification combined with posterior chamber IOL implantation in pathological eyes can result in favourable anatomic and visual outcomes in patients with macular degeneration [16, 17] glaucoma, [18–20] proliferative diabetic retinopathy [21, 22] and amblyopia [8, 23]. Additional studies would be required to further evaluate long-term complications and visual performance.

## Conclusion

As globally the population over 60 years of age continues to increase, it is estimated that the number of patients undergoing cataract surgery will keep on rising. The number of procedures performed on multi-pathological eyes will follow the same trend and it is important to provide these eyes with safe and performant solutions. Overall, findings from this study indicate good to excellent visual and refractive outcomes, with a low rate of complications up to 1 year following implantation of the CT ASPHINA 409 in healthy eyes and in eyes with concomitant pathology.

## Data Availability

The data that support the findings of this study are available from Carl Zeiss Meditec AG but restrictions apply to their availability. The data are available from Carl Zeiss Meditec AG upon reasonable request.

## Abbreviations

CDVA: Corrected distance visual acuity
CI: Confidence interval
D: Dioptre
EUREQUO: European registry of quality outcomes for cataract and refractive surgery
IOL: Intraocular lens
IOP: Intraocular pressure
ISO: International organization for standardization
logMAR: Logarithm of minimum angle of resolution
Nd:YAG: Neodymium-doped yttrium aluminium garnet
PCO: Posterior capsule opacification
SD: Standard deviation
UDVA: Uncorrected distance visual acuity
